# Microstructure and Photocatalytic Performance of BaTi_5_O_11_ Nanocrystals Synthesized via Sol-Gel Method Mediated by Organic Solvents

**DOI:** 10.3390/gels11090706

**Published:** 2025-09-03

**Authors:** Honghua Wang, Tianchen Gao, Xinyi Li, Yuci Huang, Junjie Wang, Zhixiong Huang, Dongyun Guo

**Affiliations:** 1 School of Materials Science and Engineering, Wuhan University of Technology, Wuhan 430070, China; 13343434177@163.com (H.W.); zhixionghuang@whut.edu.cn (Z.H.); 2 Ji Hua Laboratory, Foshan 528200, China

**Keywords:** BaTi_5_O_11_ nanocrystals, sol–gel method, solvents, photocatalytic performance, polyethylene glycol-200, methylene blue

## Abstract

BaTi_5_O_11_ nanocrystals were synthesized via a sol–gel method employing different organic solvents. The influence of solvent choice on microstructure and photocatalytic performance was investigated through methylene blue (MB) degradation under UV light irradiation. The monoclinic BaTi_5_O_11_ nanocrystals were successfully synthesized, where solvent selection significantly affected their grain size and Brunauer–Emmett–Teller (BET) surface area. The BaTi_5_O_11_ nanocrystals synthesized using polyethylene glycol-200 (PEG-200) exhibited the highest BET surface area (9.78 m^2^/g) and smallest average pore size (17.8 nm). The BaTi_5_O_11_ nanocrystals also displayed a larger optical bandgap (3.61 eV), attributed to pronounced quantum confinement and surface effects. Consequently, the PEG-200-derived BaTi_5_O_11_ photocatalyst achieved complete MB degradation within 30 min under UV light irradiation. This enhanced performance was attributed to the high BET surface area providing abundant active sites. Furthermore, the BaTi_5_O_11_ nanocrystal photocatalyst maintained excellent reusability and stability over four consecutive cycles.

## 1. Introduction

With the acceleration of industrialization, industrial wastewater containing a large number of organic pollutants, such as dyes, fertilizers, pesticide or surfactant molecules, is continuously discharged into water bodies [1,2,3,4]. Those organic matters are difficult for microorganisms to directly degrade in nature. Methylene blue (MB), which can be found in industrial wastewater, is a carcinogenic pollutant due to its hazardous impacts on humans, even in low concentration [5]. It is recognized that perovskite nanocrystals are efficient in the photocatalytic degradation of MB. Photocatalysis is considered as a prospective technology for the degradation of MB due to high efficiency, no secondary pollution, and environmental compatibility [6]. However, most outstanding perovskite photocatalysts, such as PrZr_x_Ti_1−x_O_3_, are hazardous to both humans and environment due to the Pb element. As typical lead-free perovskite photocatalysts, the compounds in the TiO_2_-rich BaO-TiO_2_ system, such as BaTi_4_O_9_ and BaTi_5_O_11_, have been extensively studied [7,8,9].

The BaTi_5_O_11_ compound has attracted extensive research interest due to its exceptional permittivity and low dielectric loss, which is widely applied in microwave communications [10,11]. Many researchers have attempted to synthesize the BaTi_5_O_11_ compound in a variety of ways. Recent advancements in nanofabrication techniques have also expanded its application frontiers, with hydrothermal synthesis, co-precipitation, and sol–gel methods enabling precise control over crystallinity and morphology of BaTi_5_O_11_ nanostructures [12,13,14,15,16,17]. Notably, emerging studies revealed that nanostructured BaTi_5_O_11_ exhibited remarkable photocatalytic activity, attributed to its unique crystal architecture [16]. The distorted TiO_6_ octahedra in its lattice generated spontaneous polarization that established built-in electric fields, while nanoscale dimensions shortened charge migration paths—a synergistic effect that significantly enhanced photogenerated carrier separation. Zhang et al. demonstrated 82% MB degradation using hydrothermal-synthesized BaTi_5_O_11_ nanocrystals within 30 min of UV light irradiation [15], while Yang et al. achieved 96.5% (60 min UV light irradiation) degradation efficiency through sol–gel-derived BaTi_5_O_11_ nanocrystals by tailoring their microstructures [17].

Appropriate synthetic approaches are important to the BaTi_5_O_11_ nanomaterials with designed microstructure and properties. Among various synthesis approaches, the sol–gel method presents distinct advantages for photocatalytic material engineering, including stoichiometric precision, low-temperature processing, and cost-effectiveness [17,18,19,20,21]. During sol–gel processing, parameters, such as sintering temperature, sintering time and solvent choice, decisively govern the crystallite size and morphology of BaTi_5_O_11_ nanocrystals. Solvent polarity and coordination ability fundamentally determine hydrolysis–condensation rates, thereby governing pore structure development and surface reactivity [22]. Although previous studies have established sintering temperature and time as key factors affecting crystallite size of BaTi_5_O_11_ nanocrystals [13], systematic investigations into solvent-mediated morphological control of BaTi_5_O_11_ nanocrystals are conspicuously lacking. Mehdi R. et al. [23] prepared ZnO nanocrystals via sole-gel methods with ethanol, ethylene glycol, 1,4-butanediol and PEG-600. The obtained results showed that higher viscosities and lower vapor pressures of solvents allow for more rapid growth of the ZnO nuclei, resulting in larger particles. Wang Y. et al. [24] prepared sol–gel-derived pure BaTiO_3_ particles using acetylacetone. The results showed that the product maintained stable degradation performance in environments containing inorganic anions, with a high *k* value of 5.541 × 10^−2^ min^−1^ degrading RhB. Mageste F. et al. [25] prepared SiO_2_ via the sol–gel method using methanol, ethanol, isopropanol and tert-butanol as solvents. The results showed that higher molecular weight led to larger pore size and higher specific surface area of the product, which was due to the different rates of hydrolysis and condensation reactions. In addition, the lower polarity of the solvent and the hydrogen bond combination led to larger particle size of the product, which resulted from slower nucleation. The dielectric constants of solvents also have a remarkable influence on the crystalline phase [26] solvent with high boiling point suppresses the hydrolysis of (CH_3_CH_2_O)_4_Ti, minimizes impurity formation and enhances the crystallinity of the material, resulting in a more homogeneous structure [27].

At present, there is no systematic study on the mechanisms of solvents to the synthesis of BaTi_5_O_11_ nanomaterials. Solvents not only dissolve reactants, but also have important effects on nucleation, nuclear growth, the grain production process, morphology control, etc. Choosing the appropriate solvent can optimize the reaction conditions and improve the quality and performance of the product.

In this study, BaTi_5_O_11_ nanocrystals were synthesized by the sol–gel method, and the effects of different solvents, including methanol, ethanol, 2-methoxyethanol, acetylacetone, ethylene glycol (EG) and polyethylene glycol-200 (PEG-200), on microstructure and photocatalytic MB degradation were systematically investigated. Also, a full understanding of the types of solvents and various parameters on the mechanism of regulating the synthesis process of nanomaterials, micromorphology and photocatalytic performance will provide guidance for the design of high-performance photocatalytic materials, even for other nanomaterials.

## 2. Results and Discussions

**Figure 1:**
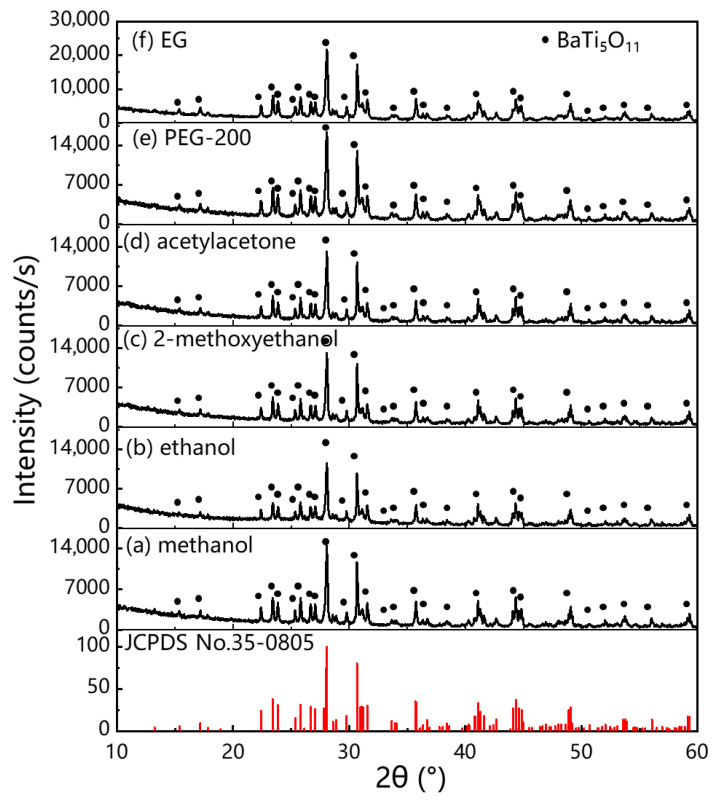
XRD patterns of BaTi_5_O_11_ samples synthesized using different solvents: (**a**) methanol, (**b**) ethanol, (**c**) 2-methoxyethanol, (**d**) acetylacetone, (**e**) PEG-200 and (**f**) EG.

Figure 1 shows the XRD results of BaTi_5_O_11_ samples synthesized using different solvents. All XRD patterns display pure crystalline phases without distinct impurity phases, and were indexed to the standard monoclinic BaTi_5_O_11_ phase (JCPDS No. 35-0805, Joint Committee on Powder Diffraction Standards). The diffraction peaks of all BaTi_5_O_11_ samples closely matched the monoclinic BaTi_5_O_11_ phase. The XRD patterns of BaTi_5_O_11_ samples prepared with different solvents were remarkably similar, indicating that all chose solvents facilitated the formation of the monoclinic BaTi_5_O_11_ phase.

**Figure 2:**
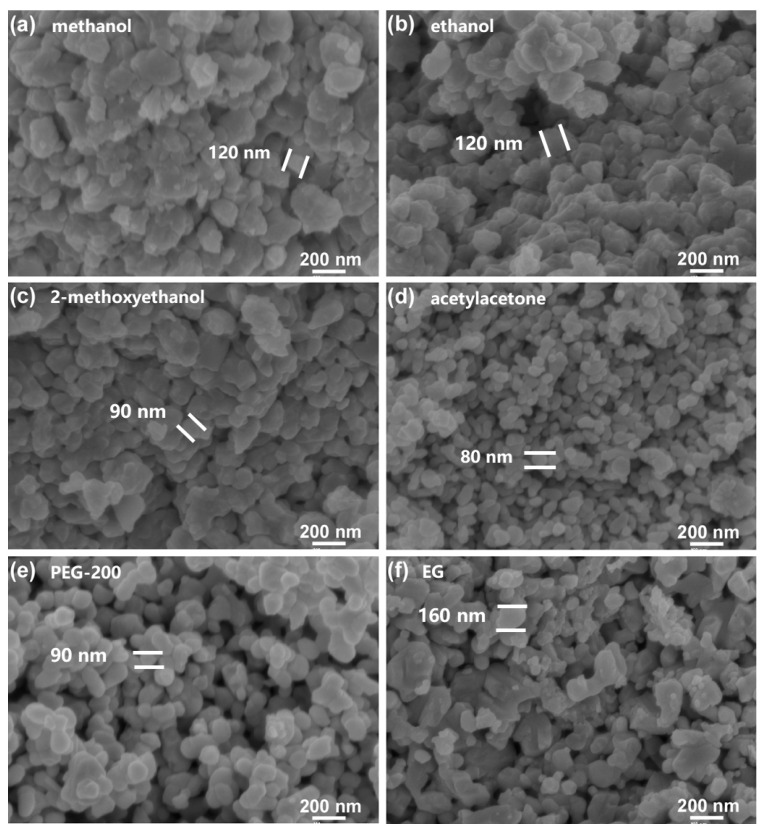
SEM patterns of the BaTi_5_O_11_ samples synthesized using different solvents: (**a**) methanol, (**b**) ethanol, (**c**) 2-methoxyethanol, (**d**) acetylacetone, (**e**) PEG-200 and (**f**) EG.

The morphologies of BaTi_5_O_11_ samples synthesized using different solvents are shown in Figure 2. The solvents with low boiling points and high volatility (methanol and ethanol) evaporated rapidly, resulting in larger BaTi_5_O_11_ grain sizes and significant agglomeration. In contrast, the solvents with higher boiling points, lower volatility and slower evaporation rates (2-methoxyethanol and acetylacetone) helped preserve the porous microstructure of the Ba–Ti gels, yielding smaller BaTi_5_O_11_ grains. Meanwhile, the solvents exhibiting even higher boiling points and substantially greater viscosity (EG and PEG-200) further slowed evaporation, which promoted nucleation over crystal growth, effectively minimizing particle agglomeration. These results demonstrated that solvents’ properties (including boiling point, viscosity, surface tension, polarity and volatility) fundamentally governed the morphology, grain size, agglomeration and specific surface area of sol–gel-derived BaTi_5_O_11_ nanocrystals [28].

**Figure 3:**
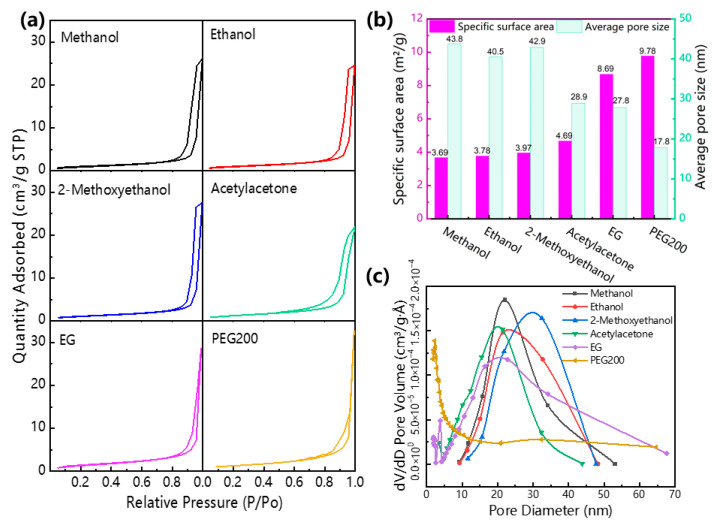
(**a**) N_2_ gas adsorption–desorption isotherms, (**b**) specific surface area and average pore size and (**c**) pore-size distribution of the BaTi_5_O_11_ nanocrystals synthesized with different solvents.

To further characterize the microstructural difference in the BaTi_5_O_11_ nanocrystals synthesized with different solvents, their pore structure and specific surface area were investigated. The BaTi_5_O_11_ nanocrystals adsorption behavior by BET method is physical adsorption, which means the adsorbed gas molecules bind to the nanocrystal with a weak van der Waals force. Figure 3a presents their N_2_ gas adsorption–desorption isotherms. Following the IUPAC (International Union of Pure and Applied Chemistry) classification, all physical absorption–desorption isotherms exhibited type IV behavior [29], with adsorption capacity increasing relative to pressure. The presence of hysteresis loops and absence of distinct adsorption plateaus indicated disordered mesoporous microstructures in all BaTi_5_O_11_ nanocrystal samples. Figure 3b displays their Brunauer–Emmett–Teller (BET) surface areas and average pore sizes. As the solvents changed from methanol, to ethanol, 2-methoxyethanol, acetylacetone, EG and PEG-200, the BET surface area increased while the average pore size decreased. The methanol-synthesized BaTi_5_O_11_ nanocrystals showed the smallest BET surface area (3.69 m^2^/g) and largest average pore size (43.8 nm), whereas PEG-200 yielded the highest BET surface area (9.78 m^2^/g) and smallest average pore size (17.8 nm). Table 1 summarizes the relevant solvent physicochemical properties. The boiling point, viscosity, volatility and surface tension of the solvents might be the primary factors influencing the microstructure of BaTi_5_O_11_ nanocrystals. This correlation was further evidenced by pore-size distributions (Figure 3c). Methanol, ethanol, 2-methoxyethanol and acetylacetone produced pores mainly between 10 and 50 nm, while PEG-200 generated significantly smaller pores of about 10 nm. However, BaTi_5_O_11_ nanocrystals prepared using EG solvent exhibited a broad pore size distribution, ranging from several nanometers to nearly 70 nm. The sol–gel method provided a relatively stable environment at a middle temperature. The rate and degree of hydrolysis and polycondensation reactions are easily affected by polarity, viscosity, boiling temperature and density. PEG-200 solvent, exhibiting even higher boiling points and substantially greater viscosity, further slowed evaporation, promoted nucleation over crystal growth, effectively minimizing particle agglomeration, which results in minimizing particle agglomeration.

**Figure 4:**
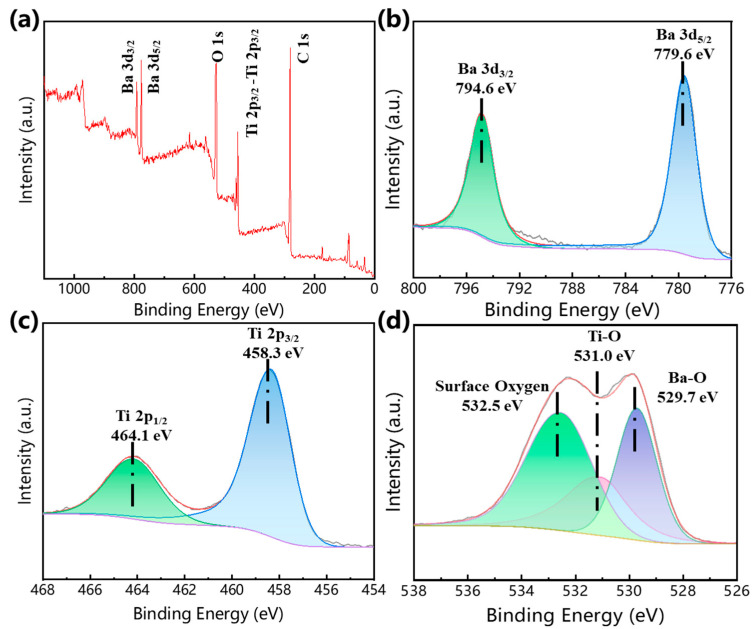
XPS spectra of BaTi_5_O_11_ nanocrystals using PEG-200 as the solvent: (**a**) XPS survey spectrum, (**b**–**d**) high-resolution XPS spectra Ba 3d, Ti 2p and O 1s, respectively.

Figure 4 presents the XPS spectra of BaTi_5_O_11_ nanocrystals synthesized using PEG-200 as the solvent. This spectroscopic tool is used for further understanding the chemical state of elements at the surface of BaTi_5_O_11_. The peak positions were calibrated by referencing the C 1s peak at 284.8 eV. The survey spectrum confirmed the presence of Ba, Ti and O elements, as shown in Figure 4a. The high-resolution spectra and their Gaussian fittings for Ba 3d, Ti 2p and O 1s are displayed in Figure 4b–d. The Ba 3d peaks resolved into Ba 3d_5/2_ (779.6 eV) and Ba 3d_3/2_ (794.6 eV) components. The Ti 2p peaks exhibited Ti 2p_3/2_ (458.3 eV) and Ti 2p_1/2_ (464.1 eV) peaks, consistent with Ti^4+^. Deconvolution of the O 1s spectrum revealed lattice oxygen of Ba–O (529.7 eV), lattice oxygen of Ti–O (531.0 eV), and a peak at 532.5 eV, attributed to surface OH^−^ groups from adsorbed water molecules on the surface of the prepared BaTi_5_O_11_ nanocrystals [24].

**Figure 5:**
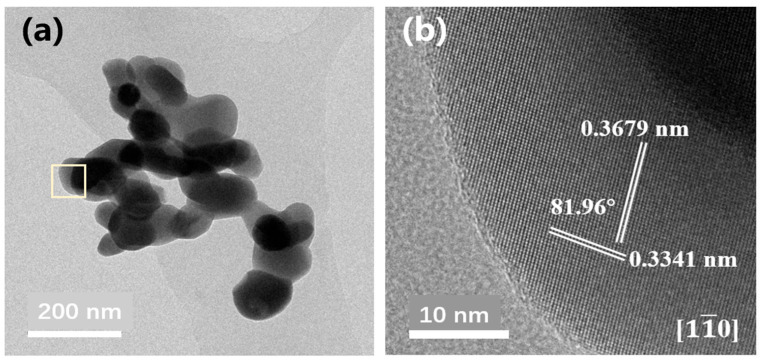
(**a**) TEM images of BaTi_5_O_11_ nanocrystals using PEG-200 as the solvent, and (**b**) its high-resolution TEM image.

Figure 5 presents the typical TEM images of BaTi_5_O_11_ nanocrystals synthesized using PEG-200 as the solvent. As shown in Figure 5a, the granular microstructure was consistent with the SEM results (Figure 2e). To assess the crystallinity of BaTi_5_O_11_ nanocrystals, the high-resolution TEM image of the nanocrystals was characterized, as shown in Figure 5b. The measured lattice spacings of 0.3341 nm and 0.3679 nm corresponded well to the (220) (0.3338 nm) and (002) (0.3727 nm) planes of the monoclinic BaTi_5_O_11_ phase, respectively. Furthermore, the measured interplanar angle of 81.96^o^ between the (220) and (002) planes was in close agreement with the theoretical value of 82.67° for the monoclinic BaTi_5_O_11_ phase (JCPDS No. 35-0805). These findings confirmed that highly crystalline BaTi_5_O_11_ nanocrystals with monoclinic crystal structure were synthesized by the sol–gel method.

**Figure 6:**
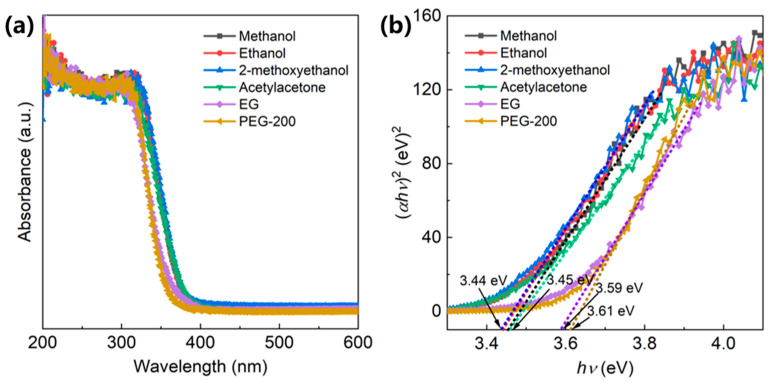
(**a**) UV–vis diffuse reflectance spectra of BaTi_5_O_11_ nanocrystals synthesized with different solvents, and their (**b**) Tauc plots of (*αhv*)^2^ vs. *hv*.

The optical properties of BaTi_5_O_11_ nanocrystals synthesized using different solvents were characterized using the UV–vis diffuse reflectance spectroscopy in the wavelength range of 200–600 nm. As shown in Figure 6a, the BaTi_5_O_11_ nanocrystals showed strong UV absorption. The optical band gap (*E_g_*) was calculated from the reflectance data using the Kubelka–Munk model of Equation (1).

(*αhν*)^*n*^ = *A* (*hν* − *E*_*g*_) (1)

where *α* is the absorption coefficient, *hν* is the photon energy and *A* is a constant. As the BaTi_5_O_11_ compound is a direct bandgap semiconductor [12], *n* is equal to two. Figure 6b shows the corresponding Tauc plots of (*αhv*)^2^ versus *hv*. The optical *E_g_* values, determined from these plots, were 3.45 eV for methanol, 3.44 eV for ethanol, 3.44 eV for 2-methoxyethanol, 3.45 eV for acetylacetone, 3.59 eV for EG, and 3.61 eV for PEG-200. The optical *E_g_* of semiconductor nanomaterials is tunable, primarily governed by quantum confinement effects and surface effects. Notably, BaTi_5_O_11_ nanocrystals synthesized using EG and PEG-200 exhibited significantly larger *E_g_* values (3.59 eV and 3.61 eV, respectively) compared to those prepared with other solvents (ranging from 3.44 to 3.45 eV). When combined with the FESEM observations (Figure 2) and specific surface area data (Figure 3), the large *E_g_* values observed for the BaTi_5_O_11_ nanocrystals using EG and PEG-200 were attributed to pronounced quantum confinement and surface effects.

**Figure 7:**
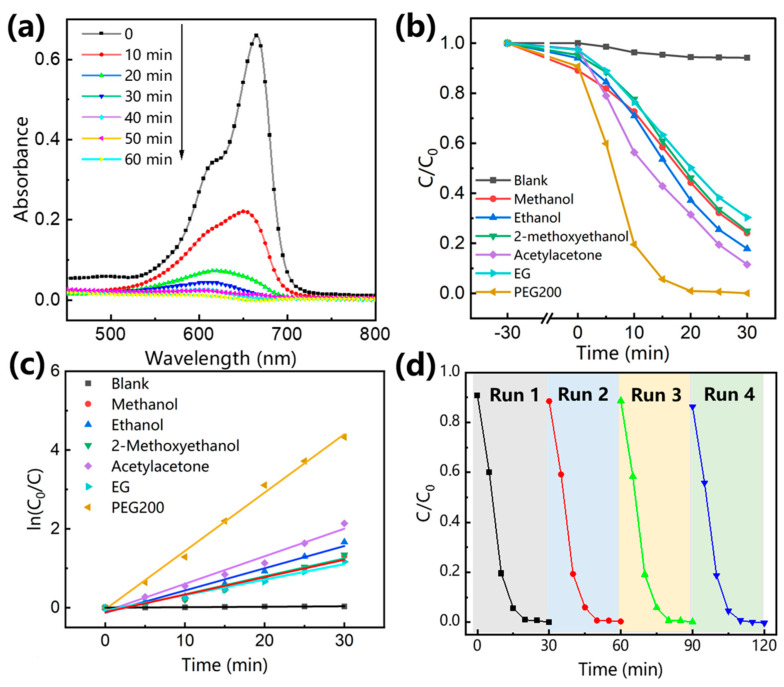
(**a**) UV–vis absorption spectra of MB solutions photodegraded at different times by BaTi_5_O_11_ nanocrystals synthesized with PEG-200 as the solvent, (**b**) time-dependent photocatalytic degradation efficiencies, and (**c**) their pseudo-first-order kinetics for MB in the presence of BaTi_5_O_11_ nanocrystals synthesized with different solvents, (**d**) photocatalytic cycling performance of BaTi_5_O_11_ nanocrystals synthesized with PEG-200 for MB degradation.

The photocatalytic performance of BaTi_5_O_11_ nanocrystals was evaluated through the photodegradation of MB under UV light irradiation. The MB concentration was determined from the UV–vis absorption intensity at its characteristic wavelength of 666 nm, which exhibited a linear relationship with concentration. Figure 7a presents the time-dependent UV–vis absorption spectra (450–800 nm) of MB solutions during degradation catalyzed by BaTi_5_O_11_ nanocrystals synthesized using PEG-200. The characteristic absorption peak at 666 nm decreased rapidly with increasing irradiation time, indicating efficient MB degradation. Figure 7b plots the normalized MB concentration (C/C_0_, where C_0_ and C represent the initial concentration and concentration at time of t, respectively) versus irradiation time for BaTi_5_O_11_ nanocrystals synthesized with different solvents. Prior to irradiation, the MB solution containing the BaTi_5_O_11_ nanocrystal photocatalyst was stirred in darkness for 30 min to establish adsorption–desorption equilibrium. The control experiments showed that MB was stable under UV light irradiation, with only 5.8% degradation occurring after 30 min in the absence of the catalyst. In contrast, the addition of BaTi_5_O_11_ nanocrystal photocatalysts led to significant MB degradation. After 30 min of UV light irradiation, the degradation efficiencies for BaTi_5_O_11_ nanocrystal photocatalysts synthesized with methanol, ethanol, 2-methoxyethanol, acetylacetone, EG and PEG-200 were 75.9%, 82.2%, 75.1%, 88.5%, 69.7% and 100%, respectively. Notably, the PEG-200-synthesized BaTi_5_O_11_ nanocrystal photocatalyst achieved complete degradation within 30 min. In our previous reports [15,16,17], the hydrothermally synthesized BaTi_5_O_11_ nanocrystals achieved 82.27% MB degradation after 30 min UV light irradiation, while the sol–gel-derived BaTi_5_O_11_ nanocrystals reached 96.5% MB degradation after 60 min UV light irradiation. The superior efficiency of the PEG-200-synthesized BaTi_5_O_11_ nanocrystals highlighted the critical influence of the solvent. The photocatalytic efficiency depends on multiple factors (catalyst properties, reaction conditions and pollutant characteristics) [30]. This study focused on the intrinsic properties of BaTi_5_O_11_ nanocrystal photocatalysts under fixed reaction conditions and pollutant parameters. The previous results (Figure 3b and Figure 6b) indicated that the solvent significantly affected the specific surface area and *E*_g_ of the BaTi_5_O_11_ nanocrystals. The PEG-200-synthesized BaTi_5_O_11_ nanocrystals exhibited the highest BET surface area, providing more active sites. However, it also showed a widened *E*_g_ (Figure 6b) due to the quantum confinement effects, potentially hindering light absorption. The experimental results demonstrated that the beneficial effect of the increased BET surface area dominated, leading to enhanced photocatalytic efficiency. Interestingly, the EG-synthesized BaTi_5_O_11_ nanocrystals also possessed a relatively large BET surface area but exhibited the lowest photocatalytic degradation efficiency (69.7%). This discrepancy was primarily attributed to differences in the solvent templating effect [23]. PEG-200, with its strong steric hindrance and templating capability, promoted the formation of a uniform, open porous microstructure. This microstructure enhanced not only the BET surface area but also dye molecule adsorption and diffusion kinetics, facilitating photocatalysis. Conversely, EG provided a weaker templating effect, often resulting in denser or agglomerated microstructures. Consequently, despite a nominally high BET surface area, insufficient exposure of active sites and restricted mass transfer in the EG-synthesized BaTi_5_O_11_ nanocrystals led to diminished photocatalytic performance.

To further study the photocatalytic kinetics of BaTi_5_O_11_ nanocrystals, their degradation reaction constant was calculated using Equation (2)

*ln*(*C*_0_/*C*) = *kt* (2)

where *t* represents the irradiation time, and *k* is the pseudo-first-order kinetic reaction rate constant. The *k* value serves as a direct indicator of photocatalytic performance, and the larger *k* value corresponds to better photocatalytic performance. Figure 7c presents degradation kinetics of the BaTi_5_O_11_ nanocrystal photocatalysts. The BaTi_5_O_11_ nanocrystal photocatalyst synthesized using PEG-200 solvent exhibited the highest *k* value of 0.28 min^−1^, while the BaTi_5_O_11_ nanocrystal photocatalyst prepared using EG solvent showed the lowest *k* value of 3.98 × 10^−2^ min^−1^. The coefficient of determination R^2^ of degradation reaction for samples were 0.994 (PEG-200), 0.976 (acetylacetone), 0.966 (ethanol), 0.958 (2-methoxyethanol), 0.958 (methanol), and 0.977 (EG), respectively. The photocatalytic cycling performance of the PEG-200-derived BaTi_5_O_11_ nanocrystal photocatalyst is shown in Figure 7d. It retained the high MB degradation efficiency of 99.7% even after four consecutive cycles, confirming the excellent reusability and stability of the BaTi_5_O_11_ nanocrystal photocatalyst synthesized using PEG-200 solvent. Based on the aforementioned results, a possible explanation for the superiority of the degradation performance of BaTi_5_O_11_ over MB synthesized using PEG-200 as solvent is proposed. The sol–gel method provided a relatively stable environment at a middle temperature. The rate and degree of hydrolysis and polycondensation reactions are easily affected by polarity, viscosity, boiling temperature and density. PEG-200 solvent, exhibiting even higher boiling points and substantially greater viscosity, further slowed evaporation, promoted nucleation over crystal growth, effectively minimizing particle agglomeration, which results in minimizing particle agglomeration. As a result, BaTi_5_O_11_ nanocrystals synthesized using PEG-200 display the highest BET surface area and smaller pores, providing more active sites to improve degradation performance. Finally, the synthesis conditions, physical characteristics, and degradation performance of BaTi_5_O_11_ synthesized using different solvents are summarized in Table 1.

## 3. Conclusions

The monoclinic BaTi_5_O_11_ nanocrystals were successfully synthesized by the sol–gel method employing different organic solvents, and their photocatalytic performance was investigated by the degradation of MB under UV light irradiation. The choice of solvent significantly influenced the grain size and BET surface area of BaTi_5_O_11_ nanocrystals. Specifically, BaTi_5_O_11_ nanocrystals synthesized using PEG-200 exhibited the high BET surface area (9.78 m^2^/g) and small average pore size (17.8 nm). The BaTi_5_O_11_ nanocrystals also demonstrated a larger optical *E*_g_ value (3.61 eV), attributed to pronounced quantum confinement and surface effects. Consequently, the PEG-200-synthesized BaTi_5_O_11_ nanocrystal photocatalyst achieved complete degradation of MB under 30 min UV light irradiation and exhibited the highest *k* value of 0.28 min^−1^. This enhanced photocatalytic performance was ascribed to the high BET surface area, which provided abundant active sites. Furthermore, the BaTi_5_O_11_ nanocrystal photocatalyst maintained excellent reusability and stability over four consecutive cycles. This study presents a simple sol–gel process for fabricating efficient BaTi_5_O_11_ nanocrystal photocatalysts, offering insights for wastewater treatment and environmental purification. Moreover, it provides fundamental guidance for understanding the role of solvents in constructing high-efficiency BaTi_5_O_11_ photocatalysts.

## 4. Materials and Methods

### 4.1. BaTi_5_O_11_ Nanocrystal Preparation

**Figure 8:**
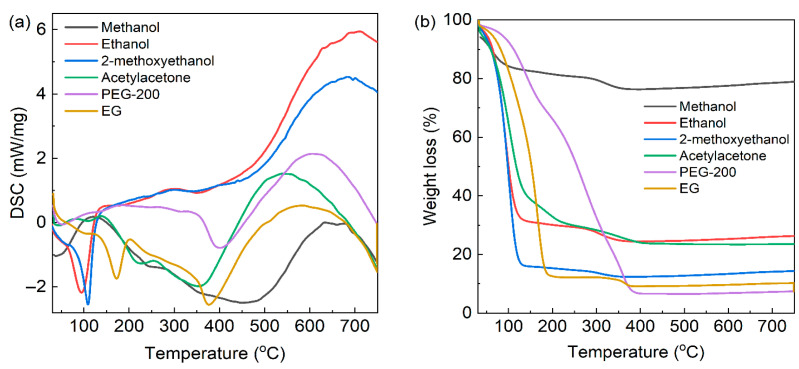
(**a**) DSC and (**b**) TG curves of BaTi_5_O_11_ precursors with different solvents.

All reagents were of analytical grade and used without further purification. Barium acetate (Ba(CH_3_COO)_2_) and tetrabutyl titanate (Ti(OC_4_H_9_)_4_) (Ba:Ti molar ratio = 1:5) were employed as Ba^2+^ and Ti^4+^ sources, respectively. The desired amount of Ba(CH_3_COO)_2_ was dissolved in acetic acid (20 mL), while Ti(OC_4_H_9_)_4_ was dissolved in the chosen solvent (80 mL, methanol, ethanol, 2-methoxyethanol, acetylacetone, EG or PEG-200). The two solutions were mixed together to form transparent Ba–Ti precursors (0.1 mol/L). Figure 1 shows the results of differential scanning calorimetry and thermogravimetric analysis (DSC-TG) for the Ba–Ti precursors with different solvents. The temperature increased from room temperature to 750 °C in N_2_ atmosphere with a heat rate of 10 °C/min. The Ba–Ti precursor prepared with methanol as the solvent exhibited an endothermic peak at a relatively low heating temperature (about 50 °C), which was attributed to the lowest boiling point and high volatility. As the boiling points of the solvents increased, the temperatures corresponding to their endothermic peaks increased. Based on Figure 8a, the baking temperatures for Ba-Ti precursors could be identified as 150 °C for those prepared with methanol, ethanol, 2-methoxyethanol, and acetylacetone, 220 °C for those prepared with EG, and 250 °C for those prepared with PEG-200. As shown in Figure 8b, the pronounced weight loss could be observed when the heating temperature approached the boiling point of each respective solvent. There was a significant difference between the weight loss of material obtained using different solvents, which was consistent with the viscosity. This phenomenon was explained by the fact that the samples synthesized using higher viscosity solvent contain more solvent in the Ba–Ti precursors.

However, the Ba-Ti precursor prepared with PEG-200 exhibited nearly linear weight loss between 100 and 380 °C. It was mainly attributed to the high viscosity of PEG-200, which resulted in slow solvent evaporation. As the temperature continued to increase, the distinct endothermic peaks appeared around 350 °C, corresponding to the pyrolysis of organic components and the formation of amorphous Ba–Ti–O powder. The TG curve also indicated a weight loss in this temperature region; consequently, 350 °C was selected as the thermal decomposition temperature. A pronounced exothermic peak near 700 °C signified the onset of BaTi_5_O_11_ crystallization, and 750 °C was chosen as the sintering temperature for the BaTi_5_O_11_ phase.

The BaTi_5_O_11_ nanocrystals were synthesized by the sol–gel method under flowing O_2_ in a quartz-tube furnace (Hangzhou Zhuochi Co., Hangzhou, China) according to the three-step heat-treatment process. In the heat-treatment process, the increase rate of temperature was 10 °C/min. These precursors were firstly baked at different temperatures (150 °C for methanol, ethanol, 2-methoxyethanol and acetylacetone; 220 °C for EG; 250 °C for PEG-200) for 180 min to evaporate the solvents, then heated at 350 °C for 180 min to obtain amorphous Ba–Ti–O powder, and finally sintered at 750 °C for 180 min to form BaTi_5_O_11_ nanocrystals. The key physicochemical properties of the solvents are listed in Table 2.

### 4.2. Material Characterization

Thermal analysis of the Ba–Ti precursors prepared with different solvents was performed with a simultaneous thermal analyzer (STA 449C, NETZSCH Group, Bavaria, Germany) to establish their optimal heat-treatment parameters. Crystal phase of the BaTi5O11 nanocrystals was characterized by an X-ray diffractometer (XRD, D8 Advance, Bruker Co., Billerica, MA, USA, Cu Kα, λ = 1.5406 Å, 40 kV and 40 mA). Morphologies were examined by field emission scanning electron microscopy (FESEM, Zeiss Ultra Plus, Jena, Germany) and transmission electron microscope (TEM, JEM-2100UHR, JEOL Ltd., Tokyo, Japan, 200 kV). Specific surface area and pore-texture properties of the BaTi5O11 nanocrystals were determined from N2 adsorption–desorption isotherms were measured by a Micromeritics ASAP 2460 analyzer (Micromeritics Instrument Co., GA, USA). Surface chemistry and elemental oxidation states of the BaTi5O11 nanocrystals were probed by X-ray Photoelectron Spectroscopy (XPS, Thermo Scientifc ESCALAB 250Xi instrument, Thermo Fisher Scientific, Inc., Waltham, MA, USA, Al Kα radiation).

### 4.3. Photocatalytic MB Degradation

The photocatalytic abilities of the BaTi_5_O_11_ nanocrystals were evaluated by monitoring the UV-driven degradation of MB. A mercury lamp (150 W) served as the UV source. Prior to irradiation, the BaTi_5_O_11_ nanocrystals (50 mg) were dispersed in aqueous MB solution (50 mL, 10 mg/L) with continuous stirring for 30 min in darkness to reach adsorption–desorption equilibrium between MB and the BaTi_5_O_11_ nanocrystal surface. When the UV light illuminated, 2 mL aliquots were taken out every 10 min, followed by centrifugation (10,000 rpm, 10 min) to remove the BaTi_5_O_11_ nanocrystal photocatalysts. The residual MB concentration in the supernatant was measured using UV–vis spectrophotometry (UV1800PC, Jinghua, Shanghai, China).

## Figures and Tables

**Table 1:** The synthesis conditions, physical characteristics and degradation performance of BaTi_5_O_11_ synthesized using different solvents.

Solvents	Methanol	Ethanol	2-Methoxyethanol	Acetylacetone	EG	PEG-200
Molecular formula	CH_4_O	C_2_H_6_O	C_3_H_8_O_2_	C_5_H_8_O_2_	(CH_2_OH)_2_	(CH_2_OH)_n_
Baking temperature (℃)	150	150	150	150	220	250
Viscosity (mPa·s, 25 °C)	0.550	1.07	1.80	1.48	17.3	22.0
Specific surface area (m^2^/g)	3.69	3.78	3.97	4.69	8.69	9.78
Average pore size (nm)	43.8	40.5	42.9	28.9	27.8	17.8
Eg (eV)	3.45	3.61	3.44	3.45	3.59	3.61
K Value (min^−1^)	4.98 × 10^−2^	9.12 × 10^−2^	5.13 × 10^−2^	0.13	3.98 × 10^−2^	0.28

**Table 2:** The physicochemical characteristics of solvents.

Solvents	Acetic Acid	Methanol	Ethanol	2-Methoxyethanol	Acetylacetone	EG	PEG-200
Molecular Formula	C_2_H_4_O_2_	CH_4_O	C_2_H_6_O	C_3_H_8_O_2_	C_5_H_8_O_2_	(CH_2_OH)_2_	(CH_2_OH)_n_
Molecular Weight	60	32	46	76	100	62	200
Boiling Point (℃)	117.9	64.7	78.3	124	141	197	250
Permittivity	6.15	32.7	25.8	16.93	23.1	37	20.5
Viscosity (mPa·s, 25 °C)	1.22	0.550	1.07	1.80	1.48	17.3	22.0
Density (g/cm^3^)	1.05	0.791	0.790	0.965	0.975	1.11	1.27
Surface Tension	31.9	20.1	22.3	27.6	27.5	48.4	49.1

## Data Availability

The data presented in this study are available on request from the corresponding authors.
